# Ethanol treatment a Non-extrusion method for asymmetric liposome size optimization

**DOI:** 10.1186/2008-2231-21-32

**Published:** 2013-04-18

**Authors:** Amir Abbas Mokhtarieh, Seyed Javad Davarpanah, Myung Kyu Lee

**Affiliations:** 1 Bionanotechnology Research Center, KRIBB, 125 Gwahak-ro, Yuseong-gu, Daejeon 305-806, Republic of Korea; 2 Department of Nanobiotechnology, University of Science and Technology, 125 Gwahak-ro, Yuseong-gu, Daejeon 305-806, Republic of Korea; 3 Applied Biotechnology Research Center, Baqiyatallah University of Medical Sciences, Tehran, Iran

**Keywords:** Gene therapy, Asymmetric liposome, Size, Extrusion

## Abstract

**Background:**

siRNA is a new tool for treatment of diseases such as cancer. However, it cannot be used directly due to rapid degradation in body fluid and blood stream; therefore, vectors are necessary for protection of siRNA against RNases and also for its precise delivery to the target cells. Since viral vector causes cancer and immune response in the host, liposomes are more preferable vectors. Liposome size is an important factor for longer circulation time. Extrusion minimizes the liposome size; however, it leads to less liposome encapsulation. Moreover, it changes structure of asymmetric liposomes.

**Findings:**

Here, ethanol treatment is introduced as a method of liposome size optimization that significantly decreases the liposome size without any effect on liposome encapsulation and its asymmetric structure formulation. For this, after liposome formation while there is some ether in solution, ethanol was added to fresh liposomes (25 and 30 percent of total liposomes volume) and liposomes were incubated at room temperature with mild agitation for 20 minutes. Finally, the extra ethanol and ether were removed by dialysis.

**Conclusion:**

Utilizing this method the liposome size was successfully decreased about 100 nm. The size of optimized liposomes (200 nm) is quite suitable for in vivo target delivery.

## Introduction

Discovery of siRNA opened a new window for treatment of diseases such as cancer. Since siRNA quickly degrades in blood stream and body fluids [1]; it needs vectors for protection and proper delivery to the target tissue. There are two different kinds of vectors for siRNA target delivery: viral systems and non-viral systems including cationic liposomes and polymers [2]. High transfection efficiency is the most important advantage of viral systems. However, its usage is limited due to inflammation and oncogenic potential of viral vectors [3,4]. These directed researchers toward the non-viral vectors such as polymers and liposomes, especially cationic liposome for nucleotide target delivery [5].

Liposomes are exposed to variety of proteins in blood stream and body fluid upon the intravenously injection which are able to increase liposomal clearance from the blood stream [6]. Liposomes removal by liver occurs due to liposome charge and size which more than fifty percent of injected liposome is being eliminated from plasma in less than one hour [7]. Reduction of liposome size either by optimization of lipid composition or physical methods of size optimizations such as extrusion can reduce the rate of liposome removal by liver and in turn leads to longer circulation time [8]. Extrusion is currently one of the most common methods of producing small unilamellar liposomes. However, it decreases the liposome encapsulation as well [9] and might change the structure of asymmetric liposomes. This method first was introduced in 1971 [10]. In order to minimize the liposome size by extrusion more than ten extrusion cycles at high trans-membrane pressures are required. However, even after extrusion all liposomes were not in the appropriate size [11]. Moreover, extrusion decreases the liposome encapsulation [9] and structure, in asymmetric cationic liposomes, which leads to non-specific cellular uptake of liposome due to changes in outer lipid formulation.

Here, we did study a method of liposome size optimization which can minimize the liposome size without any effect on encapsulation and structure of asymmetric liposome formulation.

## Material and methods

All lipids for preparing asymmetric liposomes (ALPs) in this study were purchased from Avanti Polar Lipids, Inc. (USA). Those were 1,2-distearoyl-sn-glycero-3-phosphocholine (DSPC), 1,2-dioleoyl-sn-glycero-3-phosphoethanolamine (DOPE), 1,2-distearoyl-sn-glycero-3-phosphoethanolamine-N-[methoxy(polyethyl-ene glycol)-2000] (mPEG-PE), 1,2-dioleoyl-3-dimethylammonium-propane (DODAP), 1,2-trioleoyl-3-dimethylammonium-propane (DOTAP), 1,2-dihexadecanoyl-sn-glycero-3-phosphoethanolamine (DPPE), and cholesterol (Ch). FITC-labeled siRNA were obtained from the Bioneer Corporation (Korea). The dialysis membrane with molecular weight cut-off values of 10 K Molecular weight cut off (MWCO) was purchased from Spectrum Laboratories (USA). The non-small cell lung carcinoma (NSCLC) cells A549, NCI-H322, NCI-H460, and NIH-3 T3 were obtained from ATCC (USA). Diethyl ether and ethanol were purchased from Sigma-Aldrich. All solutions were made up in Diethylpyrocarbonate (DEPC) water.

Asymmetric liposomes (ALPs) were prepared as previously explained [12]. Briefly, the inner and outer inverted micelles were prepared separately in two different test tubes. The lipid components of the outer layer were DSPC:DOPE:mPEG-PE:Ch (2:2:1:4, 1.6 μmol total) and DOTAP (0.13 μmol) only for control liposomes if indicated in text. The inner leaflet was DOTAP:DODAP:DOPE (4:5:1, 1.5 μmol total). The outer lipid film was hydrated in a mixture of 200 μl of HEPES buffer Saline (HBS) (20 mM HEPES and 150 mM sodium chloride; pH 7.5) and 120 μl ethanol (to be able to dissolve mPEG-PE); and the inner leaflet was hydrated in 150 μl of sodium citrate (150 mM, pH 4) containing 100 μg of siRNA. 600 μl and 400 μl diethyl ether was added to the outer and inner tubes respectively in order to make the outer and inner inverted micelles. Finally, the inverted micelles of outer and inner leaflets were mixed, and then diethyl ether was removed completely by nitrogen gas and dialysis with 10 K MWCO membrane pore size in HBS.

In order to minimize the liposome size by ethanol treatment method; immediately after liposome formation and evaporation of ether by nitrogen gas and before removing of ether completely by dialysis from the fresh liposomes, ethanol was added to the tubes containing fresh liposome to final concentration of 25 and 30 percent of total liposome volume and tubes were inverted several times to mix it properly and then liposomes were incubated at room temperature with moderate agitation for 20 minutes. The extra ethanol which was added for size optimization was removed by dialysis with 10 K MWCO membrane pore size in HBS.

Encapsulation efficacy of optimized ALPs after ethanol treatment was monitored by using 4% agarose gel. The optimized ALPs were loaded on the 4% agarose gel in the Tris-borate-EDTA (TBE) buffer with 0.5 μg/ml ethidium bromide in the absence and presence of 1% nonidet-P40 (NP40), non-optimized ALPs were used as control. The siRNA bands were visualized with a gel documentation system using an ultraviolet transilluminator (Bio-Rad, USA). The siRNA band intensities were determined by means of densitometric analyses using the Bio2D program (Vilber Lourmat, France).

Liposome aggregation assay was carried out by mixing of 30 μl of optimized ALPs with 70 μl fetal bovine serums (FBS) and mixing of 30 μl of non-optimized ALPs with 70 FBS as control. Then samples were incubated at 37°C for 8 hours. Turbidity of samples was measured using 550 nm with a multi label plate reader (PerkinEl-mer VICTOR 3 1420, USA).

Cellular uptake was done to check the stability of asymmetric structure of optimized ALPs after size treatment. The NSCLC cells (A549, NCI-H322 and NCI-H460) were cultured in micro-slide eight-well microscopy chambers (ibidi 8 well plates, Germany) in DMEM-FBS. Then cells were treated with optimized ALPs (containing FITS-siRNA) and control liposome which was prepared by adding of DOTAP (0.13 μmol) to the outer layer. After 1 day of incubation, the cells were rinsed twice in PBS and fixed with 4% formaldehyde in PBS at room temperature for 10 min. The cellular uptake of FITC-siRNA was monitored with a confocal microscope.

## Results

**Figure 1:**
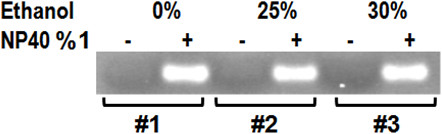
**Ethanol treatment and siRNA encapsulation.** Optimized ALPs with 30% and 25% ethanol and control non-optimized ALPS (0%) were loaded in adjacent wells in absence and presence of 1% NP40 to evaluate siRNA encapsulation.

The effect of ethanol treatment on ALPs siRNA encapsulation was evaluated by gel electrophoresis assay. Optimized liposomes showed more than 90% siRNA encapsulation, comparable with control non-optimized ALPs (Figure 1), which means ethanol does not have any effect on liposome encapsulation. However, the size of optimized ALPs dramatically was decreased from 365.7 nm to 225 and 212 nm depending on the ethanol concentration (Table 1). The optimized ALPs size was much smaller after ethanol treatment.

**Table 1:** Effect of ethanol treatment on liposome size

	**Size (nm)**	**Zeta potential (mV)**	**Polydispersity (e-1)**
Control Liposome	365.7 ± 41.3	4.97 ± 1.9	2.664 ± 0.15
Treated with 25% Ethanol	255.0 ± 52.2	1.15 ± 2.0	2.242 ± 0.46
Treated with 30% Ethanol	212.5 ± 24.0	−3.25 ± 1.4	2.463 ± 0.19

In order to evaluate the effect of ethanol treatment on the structure of outer layer of optimized liposome which is important in liposome interaction with serum proteins and elimination of liposomes from the blood, aggregation assay and cellular uptake study were performed.

**Figure 2:**
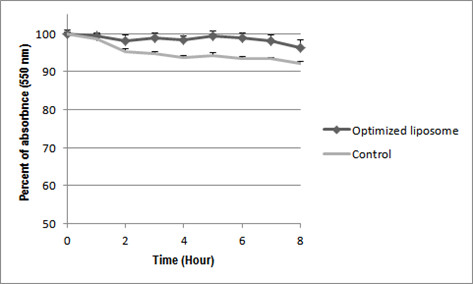
Liposome aggregation assay (P < 0.05).

Optimized liposome with ethanol, the same as control non-optimized ALPs, did not have any significant change in turbidity during 8 hours incubation in presence of FBS (Figure 2), which means the optimized ALPs has no interaction with the serum protein, quite similar to control no-optimized ALPs.

**Figure 3:**
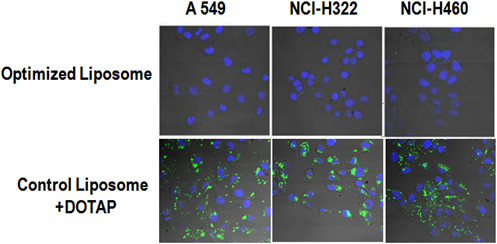
**Cellular uptake of optimized ALPs.** Control non-optimized ALPs containing DOTAP in outer layer showed a high cellular uptake (down). However, optimized liposome had no cellular uptakes which certify might not be DOTAP in outer leaflet (up).

Treatment of NSCLC cells with optimized ALPs and non-optimized-ALPs containing DOTAP in outer layer as control were done in order to evaluate the possible replacement of cationic lipids of inner layer, DOTAP, with any of lipids in outer layer which can change the structure and properties of ALPs after size optimization with ethanol. As the results show, the optimized liposomes has no penetration in none of the NSCLC cells (Figure 3, up), similar to non-optimized ALPs [12]; however the control liposomes containing DOTAP in outer layer penetrated in all of the cells (Figure 3, down).

## Discussion

Asymmetric liposomes using cationic lipid presents 90% siRNA encapsulation [12]. The asymmetric structure of liposomes eliminates non-specific liposomal cellular uptake arising from the positive charge of cationic lipids [12]. Cationic lipids are essential and inevitable in liposome formulation for higher siRNA encapsulation whereas the asymmetric structure is important for eliminating of non-specific targeting. Therefore, it is important to optimize and minimize the asymmetric liposome size by a method which has no effect on the asymmetric structure of liposomes and moreover makes it in appropriate size for longer circulation time. Here, we introduce ethanol treatment as a proper method of liposome size optimization that reduces the liposome size without any specific effect on the ALPs structure and properties.

Treatment of liposomes by ethanol to final concentration of 25 and 30 percent remarkably decreases the liposome size, about 100 nm from 365.7 nm to 225 nm or 212.5, and zeta potential, from 4.97 ± 1.9 to −3.25 ± 1.4, depending on the ethanol concentration (Table 1); however, it did not decrease the siRNA encapsulation (Figure 1). It is critical to add ethanol before complete ether removal by dialysis since after ether removal; ethanol treatment decreases the liposome encapsulation to 65–70 percent (Data not shown).

One of the major reasons of liposomes clearance from the blood circulation is liposome charge. Positive charge of cationic liposomes not only causes quick liposome elimination from blood circulation, due to interaction of liposomes with serum proteins, but also it increases non-specific liposome removal by cell. However, asymmetric cationic liposomes having positive charge only in inner layer showed high siRNA encapsulation and no non-specific cellular uptake [12]. Any change in asymmetric structure of optimized ALPs after ethanol treatment might leads to changes in liposome surface charge and in turn leads to higher interaction of optimized ALPs with the serum proteins. However, turbidity of optimized ALPs did not changed during 8 hours incubation similar as turbidity of control non-optimized ALPs (Figure 2). It proves that the ethanol treatment might not have any significant effect on the liposome structure so that both optimized and control ALPs show the same results.

Cellular uptake study was done with control liposomes containing DOTAP in outer layer (0.13 μmol, equal with 20 percent of total DOTAP in inner layer) to induce positive charge on the liposome surface. The results of cellular uptake study (Figure 3) showed no cellular uptake only for optimized liposome. This was another proof that there might not be any change in the asymmetric liposomes structure due to ethanol treatment, since the replacement of inner layer DOTAP with any lipids of outer layer could induce positive charge on the liposome surface and increase cellular uptake consequently.

## Conclusion

In conclusion, ethanol treatment is a simple and an appropriate method of liposome size optimization which decreases the liposome size effectively without any effect on siRNA encapsulation and properties.

## Competing interests

The authors declare that they have no competing interests.

## Authors’ contributions

MKL just has been an advisor and research has done in his lab. AAM has done all research work and experiments. SJD has done manuscript writing, and also let the first author, AAM, to use his lab and facilities for some experimental work related to the manuscript when the author was in Iran. All authors read and approved the final manuscript.

## References

[B1] Morrissey DV Lockridge JA Shaw L Potent and persistent in vivo anti-HBV activity of chemically modified siRNA Nat Biotechnol 2005 23 1002 1007 10.1038/nbt1122 16041363

[B2] Varmus H Retroviruses Science 1998 40 1427 1435 10.1126/science.3287617 3287617

[B3] Kawasaki S Hashida M Targeted delivery system of small interfering RNA by systemic administration Drug Metab Pharmacokinet 2007 22 142 151 10.2133/dmpk.22.142 17603214

[B4] Donahue RE Kessler SW Bodine D Helper virus induced T cell lymphoma in nonhuman primates after retroviral mediated gene transfer J Exp Med 1992 176 1125 1135 10.1084/jem.176.4.1125 1383375 PMC2119385

[B5] Sorgi FL Bahattacharya S Huang L Protamine sulfate enhance lipid-mediated gene transfer Gene Ther 1997 4 961 8 10.1038/sj.gt.3300484 9349433

[B6] Bonte F Julaino RL Lnteractions of liposomes with serum proteins Chem Phys Lipids 1986 40 359 72 10.1016/0009-3084(86)90079-4 3742678

[B7] Chonn A Semple SC Cullis PR Association of blood proteins with large unilamellar liposomes in vivo. Relation to circulation lifetimes J Biol Chem 1992 267 18759 65 1527006

[B8] Parr MJ Masin D Cullis PR Bally MB Accumulation of liposomal lipid and encapsulated doxorubicin in murine Lewis lung carcinoma: the lack of beneficial effects by coating liposomes with poly(ethy1ene glycol) J Pharmacol Exp Ther 1997 280 1319 27 9067319

[B9] Jousma H Talsma H Spies F Joosten JGH Junginger HE Crommelin DJA Characterization of liposomes. The influence of extrusion of multilamellar vesicles through polycarbonate membranes on particle size, particle size distribution and number of bilayers Int J Pharm 1987 35 214 63

[B10] Johnson SM Bangham AD Hill MW Korn ED Single bilayer liposomes Biochim Biophys Acta 1971 233 820 826 10.1016/0005-2736(71)90184-2 4107123

[B11] Ohsawa T Miura H Harada K A novel method for preparing liposome with a high capacity to encapsulate proteinous drugs: freeze-drying method Chem Pharm Bull 1984 32 2442 2445 10.1248/cpb.32.2442 6488413

[B12] Mokhtarieh AA Cheong S Kim S Chung BH Lee MK Asymmetric liposome particles with highly efficient encapsulation of siRNA and without nonspecific cell penetration suitable for target-specific delivery BBA-biomembrane 1818 2012 1633 41 10.1016/j.bbamem.2012.03.016 22465072

